# Calculated Parameters of Thyroid Homeostasis: Emerging Tools for Differential Diagnosis and Clinical Research

**DOI:** 10.3389/fendo.2016.00057

**Published:** 2016-06-09

**Authors:** Johannes W. Dietrich, Gabi Landgrafe-Mende, Evelin Wiora, Apostolos Chatzitomaris, Harald H. Klein, John E. M. Midgley, Rudolf Hoermann

**Affiliations:** ^1^Medical Department I, Endocrinology and Diabetology, Bergmannsheil University Hospitals, Ruhr University of Bochum, Bochum, Germany; ^2^Ruhr Center for Rare Diseases (CeSER), Ruhr University of Bochum, Bochum, Germany; ^3^Ruhr Center for Rare Diseases (CeSER), Witten/Herdecke University, Bochum, Germany; ^4^Zentrum für Unfallchirurgie, Orthopädie und Wirbelsäulenchirurgie, HELIOS Klinikum Schwelm, Schwelm, Germany; ^5^North Lakes Clinical, Ilkley, UK; ^6^Department of Nuclear Medicine, Klinikum Luedenscheid, Luedenscheid, Germany

**Keywords:** thyroid hormones, homeostasis, SPINA-GT, SPINA-GD, set point, feedback control, thyroid’s secretory capacity, sum activity of peripheral deiodinases

## Abstract

Although technical problems of thyroid testing have largely been resolved by modern assay technology, biological variation remains a challenge. This applies to subclinical thyroid disease, non-thyroidal illness syndrome, and those 10% of hypothyroid patients, who report impaired quality of life, despite normal thyrotropin (TSH) concentrations under levothyroxine (L-T4) replacement. Among multiple explanations for this condition, inadequate treatment dosage and monotherapy with L-T4 in subjects with impaired deiodination have received major attention. Translation to clinical practice is difficult, however, since univariate reference ranges for TSH and thyroid hormones fail to deliver robust decision algorithms for therapeutic interventions in patients with more subtle thyroid dysfunctions. Advances in mathematical and simulative modeling of pituitary–thyroid feedback control have improved our understanding of physiological mechanisms governing the homeostatic behavior. From multiple cybernetic models developed since 1956, four examples have also been translated to applications in medical decision-making and clinical trials. Structure parameters representing fundamental properties of the processing structure include the calculated secretory capacity of the thyroid gland (SPINA-GT), sum activity of peripheral deiodinases (SPINA-GD) and Jostel’s TSH index for assessment of thyrotropic pituitary function, supplemented by a recently published algorithm for reconstructing the personal set point of thyroid homeostasis. In addition, a family of integrated models (University of California-Los Angeles platform) provides advanced methods for bioequivalence studies. This perspective article delivers an overview of current clinical research on the basis of mathematical thyroid models. In addition to a summary of large clinical trials, it provides previously unpublished results of validation studies based on simulation and clinical samples.

## Introduction

Thanks to the advent of sensitive assays for TSH and free thyroid hormones, the diagnosis of classical forms of overt hypothyroidism and hyperthyroidism has become a straightforward task (1). Differential diagnosis may still be difficult, however, in some cases with subclinical forms of thyroid failure (2, 3), hypothalamic or pituitary dysfunction (4), and in situations of allostatic load, e.g., starvation and non-thyroidal illness syndrome (NTIS) (2, 5, 6). A therapeutic challenge arises from the fact that current standard treatment of hypothyroidism with levothyroxine (l-T4) fails to raise the quality of life (QoL) in patients to a level observed in a normal population (7). Rather, they display symptoms that are compatible with either hypothyroidism and hyperthyroidism, and a fraction of 5–15% of hypothyroid patients on l-T4 replacement continue to complain about impaired QoL, despite documented biochemical euthyroidism as defined by reference intervals (7, 8).

Reasons for low health-related QoL in treated hypothyroidism may include inadequate dosage of substitution therapy with l-T4, inadequate treatment modality, systemic sequelae of thyroid autoimmunity, concomitant other autoimmune diseases, and psychological phenomena, especially in form of a nocebo effect (7). Additionally, low reported QoL might ensue from some selection bias, since in most countries thyroid disease is treated by primary care physicians, who may refer “difficult cases” to academic centers, and since most functional thyroid disorders are diagnosed because patients report elements of lower QoL (9–11). According to the topic of this perspective article, we will focus our subsequent considerations to the former two possible mechanisms.

Inadequate treatment modality refers to potential adverse effects of monotherapy with l-T4, e.g. in a subgroup of hypothyroid patients, who are affected by reduced deiodination due to polymorphic variants with lower enzyme activity (12, 13). In this group, additional replacement with l-T3 (and also, possibly, low doses of other classical and non-classical thyroid hormones) may be beneficial. Due to disruption of the thyroid-mediated TSH–T3 shunt (14, 15), inefficient conversion of T3 from T4 may also arise in the subgroup of l-T4-treated athyreotic patients (16, 17). Narrow individual tolerance to hormone concentrations around the personal set point of thyroid homeostasis may also contribute to considerable variation in the treatment response (18–21). These observations have stimulated a recent debate, whether universal reference ranges for TSH and peripheral thyroid hormones are appropriate (14). Improved diagnostic efficiency has also been observed using multivariate analysis rather than the conventional univariate approaches (22). Based on recent research, we and others have propagated a more comprehensive systems-based approach. This includes the use of homeostatically defined structure parameters (6). Mathematical modeling of pituitary–thyroid feedback control has delivered functional insights beyond the scope of univariate reference ranges (14, 20, 23, 24).

This perspective article gives an overview of current methodology and established and possible future applications of modeling-based diagnostic investigation *in vivo*.

## Applying Cybernetic Models of Thyroid Homeostasis

Over the past 60 years, a plethora of mathematical or simulative models of pituitary–thyroid interaction has been published (6, 14). Only a small subset, however, has been translated into applications for clinical decision-making or research (beyond the scope of modeling itself). These modeling platforms include the logarithmic standard model of thyroid homeostasis (25), compartment analytical models, which were originally developed at the Biocybernetics Laboratory of the University of California-Los Angeles (subsequently referred to as UCLA platform) (26–31), non-linear models combining Michaelis–Menten kinetics in the feedforward path and non-competitive inhibition in the feedback direction (aka MiMe-NoCoDI models) (32, 33), and a so-called “minimal model” that combines Michaelis–Menten kinetics with a logarithmic model of hypothalamic–pituitary function (20, 23, 24). Thanks to both sufficient empirical foundation and some physiological justification, models derived from these platforms are able to deliver meaningful measures of homeostatic function. Where biochemical knowledge is (or was) insufficient for the development of well-justified models, simple equations, e.g., ratios, have been introduced to deliver an estimate for basic processes of conversion or signal transduction.

## Applications of the UCLA Platform

This family of models is based on separation of source and sink organ components, implemented as at least three source (organ) and three sink (distribution and elimination) subsystems. Dating back in its origin to 1966 (34–36), it was successively improved to incorporate current findings of basic and clinical research. The most recent implementations of this platform combine Michaelis–Menten kinetics (deiodination) with a three-parameter time-delay model (thyroid), a negative exponential model for feedback inhibition of TSH release, and a non-linear description of plasma protein binding (26–31).

Models of this platform were applied to pharmacokinetic (PK) and pharmacodynamic questions concerning substitution therapy with l-T4 (37). By mathematical modeling and computer simulations, it could be demonstrated that for the majority of hypothyroid patients standard l-T4-only therapy should be sufficient to reach normal triiodothyronine tissue concentrations (28, 29), but that substitution with l-T3 may be beneficial to reduce the withdrawal period before ^131^I remnant ablation in patients with thyroid cancer (26).

Additionally, this platform paved the way for the development of an improved protocol for bioequivalence studies. Thyroid hormones are critical dosage drugs, i.e., small changes in concentration may exert major metabolic effects, and the absorption rate is highly sensitive to multiple influencing factors including meals, coffee, concomitant medication, and gastrointestinal disease (38). Moreover, l-T4 preparations of different brands cannot be considered bioequivalent (39, 40). Traditionally, bioequivalence is assessed by PK studies as required by the FDA and other regulatory authorities. Standard protocols are faced with the problem that they ignore the existence of functional feedback in healthy volunteers, however. Models based on the UCLA platform delivered an improved baseline correction method that is less prone to this kind of interference (27, 28).

## Measures of Thyroid Function and Peripheral Hormone Metabolism

Circulating T4 is actively taken up by cells and biologically activated by enzymatic monodeiodination before exerting (mostly genomic) intracellular effects. The molar T3/T4 ratio may therefore serve as a simple measure of deiodinase activity and conversion efficiency. Numerous studies investigated the T3/T4 ratio in various conditions. They found it to be increased in iodine deficiency (41) and other settings of hyperdeiodination (42–44) – possibly accompanied by intrathyroidal hypoiodination and representing an iodine recovery mechanism – and to be decreased in NTIS (45, 46), central hypothyroidism ensuing from thyrotropic insufficiency (47, 48), congenital thyroid hypoplasia (47), treatment with propranolol (49) and, compared with cases of true hyperthyroidism, in the acute phases of postpartum thyroid dysfunction, subacute, and painless thyroiditis (50–53). It is increased in Graves’ disease compared to multinodular toxic goiter and toxic adenoma (51) and decreased in athyreotic patients receiving substitution therapy with l-T4 (54). An observation study found a negative correlation of T3/T4 ratio with age and positive correlation with serum selenium concentration (55). A report on strong negative correlation between FT4 index and T3/T4 ratio remains questionable, since the results were not corrected for spurious correlations (56).

Other measures related to conversion are FT3/reverse T3 (rT3) ratio, an estimate for the proportion of step-up to step-down deiodination, and 3,5-diiodothyronine (3,5-T2)/FT3 ratio. The former parameter is decreased in NTIS (TACITUS), while the latter is increased (57).

However, the simple ratios are conceptually incompatible with known kinetic properties of enzyme-mediated processes, as they wrongly assume linear relationships (6). Reference ranges for ratios are also more difficult to define than for non-fractions (58). These inherent deficiencies made it necessary to derive more robust structure parameters that describe the behavior of transfer elements in homeostatic models (33, 59). The novel parameters are based on the MiMe-NoCoDI platform, i.e., Michaelis–Menten functions and PK data to deliver a structure parameter inference approach (SPINA) that provides non-linear estimates of signal transduction (32).

To implement this approach, we estimated the sum activity of peripheral deiodinases (${Ĝ}_{\text{D}}$ or SPINA-GD), which reflects the maximum stimulated activity of step-up deiodination. It is calculated with

$${Ĝ}_{\text{D}}=\frac{\beta_{31}\left(K_{\text{M}1}+\left[\text{F}{\text{T}}_{4}\right]\right)\left(1+K_{30}\left[\text{TBG}\right]\right)\left[\text{F}{\text{T}}_{3}\right]}{\alpha_{31}\left[\text{F}{\text{T}}_{4}\right]}$$

from equilibrium concentrations of FT4, FT3, and PK constants (Table 1) (32, 60). A simpler version employs the concentration of total T3 with

$${Ĝ}_{\text{D}}=\frac{\beta_{31}\left(K_{\text{M}1}+\left[\text{F}{\text{T}}_{4}\right]\right)\left[\text{T}{\text{T}}_{3}\right]}{\alpha_{31}\left[\text{F}{\text{T}}_{4}\right]}.$$

**Table 1:** **Standard parameters used by the equations for SPINA-GT, SPINA-GD, and Jostel’s TSH index (6, 32, 60)**.

**Symbol**	**Explanation**	**Value**
α_T_	Dilution factor for thyroxine	0.1 L^−1^
β_T_	Clearance exponent for T4	1.1e–6 s^−1^
*D*_T_	EC_50_ for TSH	2.75 mIU/L
*K*_41_	Dissociation constant of T4 at thyroxine-binding globulin	2e10 L/mol
*K*_42_	Dissociation constant of T4 at transthyretin	2e8 L/mol
α_31_	Dilution factor for triiodothyronine	0.026 L^−1^
β_31_	Clearance exponent for T3	8e−6 s^−1^
*K*_M1_	Dissociation constant of type 1 deiodinase	500 nmol/L
*K*_30_	Dissociation constant of T3 at thyroxine-binding globulin	2e9 L/mol
[TBG]	Standard concentration of thyroxine-binding globulin	300 nmol/L
[TBPA]	Standard transthyretin concentration	4.5 μmol/L
β	Correction coefficient of logarithmic model	0.1345

*Dilution factors are defined as the reciprocal of apparent volume of distribution (*V*_D_)*.

The reference range for SPINA-GD is typically between 20 and 60 nmol/s (57), with some dependence on the assays used. Since the dissociation constant of type 1 deiodinase is beyond physiological plasma concentrations of FT4, SPINA-GD is nearly linear in the euthyroid range, so that it has similarities to the T3/T4 ratio. Its non-linear properties are advantageous especially in cases of high FT4 concentrations.

The thyroid’s secretory capacity (${Ĝ}_{\text{T}}$ or SPINA-GT), also referred to as thyroid output or thyroid capacity, provides an estimate for the maximum secretion rate of the thyroid gland under stimulated conditions. It is defined with

$${Ĝ}_{\text{T}}=\frac{\beta_{\text{T}}\left(D_{\text{T}}+\left[\text{TSH}\right]\right)\left(1+K_{41}\left[\text{TBG}\right]+K_{42}\left[\text{TBPA}\right]\right)\left[\text{F}{\text{T}}_{4}\right]}{\alpha_{\text{T}}\left[\text{TSH}\right]}$$

as a function of equilibrium concentrations of TSH, free T4, and constants or measured values for dissociation, protein binding, distribution, and elimination (Table 1) (32, 60). A simpler version utilizes total T4 concentration with

$${Ĝ}_{\text{T}}=\frac{\beta_{\text{T}}\left(D_{\text{T}}+\left[\text{TSH}\right]\right)\left[\text{T}{\text{T}}_{4}\right]}{\alpha_{\text{T}}\left[\text{TSH}\right]}.$$

The reference range is usually between 1.4 and 8.7 pmol/s (57).

**Figure 1:**
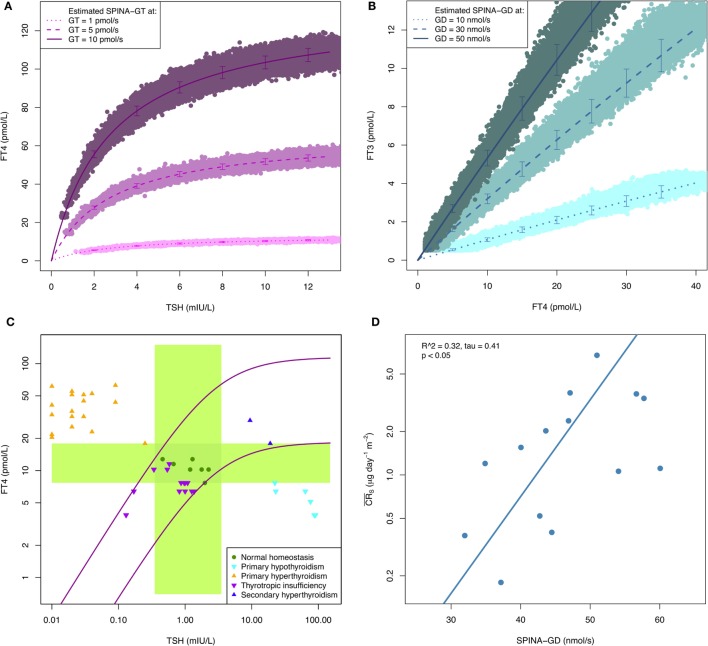
**(A,B)** Reliability of SPINA-derived parameters is higher than that of measured hormone concentrations. Shown are results of Monte Carlo evaluation of SPINA-GT and SPINA-GD based on simulated imprecise hormone assays. Hormone concentrations were modeled in SimThyr 4.0 (64) with different pre-defined values for GT and GD, respectively. Subsequently, absolute hormone levels were converted to measurements by means of an S script (see supplementary code for an introductory example) that injected additive and multiplicative noise, in order to get vendor-reported concentration-dependent coefficients of variations (CV) (65, 66). The lines show mean ± SD of hormone concentrations predicted by structure parameters calculated from simulated noisy measurements. CVs as markers for measurement reliability (67) of SPINA-GT and SPINA-GD are below 10% in all cases, although CVs of corresponding hormone assays exceed 20% in low concentrations. **(C)** SPINA-GT is sensitive for thyroid disorders of primary origin and specific with respect to secondary dysfunction. The plot shows distribution of hormone concentrations in certain primary and secondary thyroid conditions compared to normal percentiles of SPINA-GT. The green crossing rectangles define univariate reference ranges for TSH and FT4, respectively. The purple lines represent FT4 concentrations at the 2 and 97% percentiles of SPINA-GT. Data from RUBIONERVE (registration number 4905-14 at RUB ethics committee) and NOMOTHETICOS studies (UTN U1111-1122-3273, ClinicalTrials.gov ID NCT01145040). **(D)** SPINA-GD is an estimate for deiodination. Shown is correlation between SPINA-GD and conversion rate in slow tissue pools. Data from Pilo et al. (63).

*In silico* evaluation with Monte Carlo simulations demonstrates that both SPINA-GT and SPINA-GD can be sufficiently reliably estimated, despite limited accuracy of laboratory assays (Figures 1A,B). *In vivo* validation confirmed that SPINA-GT is able to clearly differentiate between euthyroidism and functional thyroid disorders of primary origin (32, 61, 62). However, unlike TSH, it is unaffected by hypothalamic–pituitary dysfunction (Figure 1C; Table S1 in Supplementary Material). This translates to high specificity in thyroid disorders of secondary or tertiary origin. Physiologically, SPINA-GD correlates with the conversion rate of slow tissue pools (Figure 1D), as determined by isotope-based measurements in healthy volunteers (63).

SPINA-GT and SPINA-GD have been validated in numerous clinical trials. In a retrospective comparison with normal controls, SPINA-GT was significantly elevated in patients with toxic adenoma, Graves’s disease, and even euthyroid diffuse and nodular goiter and significantly reduced in autoimmune thyroiditis (32). In the same study, it had a higher specificity for hyperthyroidism in toxic adenoma than TSH, FT4, or FT3 concentrations (32). A small trial with 20 healthy volunteers revealed the re-test reliability of SPINA-GT to be higher than that of every other parameter (6, 32). SPINA-GT was also shown to correlate with thyroid volume (32) and creatinine clearance (68).

Multiple trials demonstrated SPINA-GD to be reduced in NTIS (57, 69–71). One of these trials also reported that SPINA-GD predicts postoperative atrial fibrillation and correlates to age, total atrial conduction time (PA-TDI interval), as well as to concentrations of B-type natriuretic peptide (BNP) and 3,5-T2 (57). Two large trials together covering >3,500 participants independently revealed SPINA-GD to correlate with TSH concentrations and to be significantly reduced after initiation of substitution therapy with l-T4 (16, 57, 72). Strong correlation with TSH levels seems to depend on the presence of residual thyroid tissue, since it was preserved in patients with autoimmune thyroiditis, but lacking in a cohort with thyroid cancer after surgery and radioiodine ablation (17). Conversely, FT3 concentrations correlated with l-T4 supply in treated cancer patients, while they remained constant over a broad range of SPINA-GT or l-T4 dosage in groups with remaining thyroid tissue. These observations suggest the existence of a thyroid-mediated TSH–T3 shunt, which might represent a compensatory mechanism, mitigating the effects of decreasing thyroid output in onset hypothyroidism (15, 17). In patients on l-T4 replacement therapy, SPINA-GD was an independent predictor of l-T4 dose (73).

If confirmed by sufficiently powered clinical trials, possible future applications of the SPINA methodology might include differential diagnosis of primary functional thyroid disorders from dysregulations of secondary or tertiary origin or from thyrotropic adaptation, i.e., transient alterations of TSH concentrations in cases of NTIS (5, 60), screening for iodine deficiency, and identification of patients who would benefit from additional substitution therapy with l-T3 (12, 13).

## Estimated Parameters for Pituitary Function

Jostel’s TSH index (TSHI) was introduced as a quantitative marker for pituitary thyrotropic function (74). Based on the logarithmic standard model of thyroid homeostasis (25), it is calculated as

$$\text{TSHI}=\ln\left(\left[\text{TSH}\right]\right)+\beta \left[\text{F}{\text{T}}_{4}\right]$$

from measured concentrations of TSH and free T4 and a correction coefficient β (Table 1). The TSHI has been calibrated in a large sample of >9,500 subjects with and without anterior pituitary insufficiency. A *z*-transformed version of the parameter was defined as standardized TSH index (sTSHI) that results with

$$\text{sTSHI}=\frac{\text{TSHI}-2.7}{0.676}$$

from mean (2.7) and SD (0.676) of the TSHI in a normal population. Accordingly, its reference range is between −2 and +2. In the original validation study, gonadotropic insufficiency and lower peak concentrations of growth hormone and cortisol in pituitary stimulation tests were associated with significantly diminished TSHI (74). Recently, it was demonstrated that the TSHI is also reduced in patients with NTIS and thyrotropic adaptation (69).

Another estimate for thyrotropic function, the thyrotroph thyroid hormone resistance index (TTSI, also referred to as thyrotroph thyroxine resistance index or TT4RI), results with

$$\text{TTSI}=\frac{100\left[\text{TSH}\right]\left[{\text{FT}}_{4}\right]}{l_{u}}$$

from equilibrium concentrations of TSH and free T4 and the upper limit of the reference interval of FT4 (*l*_u_) (75). This screening parameter is elevated in cases of resistance to thyroid hormone due to mutations in the THRB gene (RTH Beta, Refetoff syndrome) (75). It may also be a valuable marker for monitoring central response to substitution therapy with triiodothyroacetate (TRIAC) in RTH beta (76). In a large cohort of twin pairs TTSI was strongly influenced by genetic factors (77). A variant of the TTSI (without correction for the upper limit of the reference range) was significantly increased in offspring from long-lived siblings compared to their partners (78). This observation suggests slight resistance to thyroid hormone to be beneficial with respect to longevity.

## Reconstructing the Set Point of Thyroid Homeostasis

Intra-individual variation of TSH and T4 concentrations is considerably lower than inter-individual variation (18, 21). This observation gave rise to the set point theory of thyroid homeostasis, i.e., to the assumption that serum levels of TSH and FT4 are controlled to match a personal, genetically encoded reference. The region around the individual set point is the obvious target for substitution therapy with l-T4. Unfortunately, however, the location of the set point is unknown and inaccessible in the situation of hypothyroidism (6, 19). It may also vary in thyroid health, l-T4 treatment (79) and NTIS/TACITUS (2, 5, 80, 81). Recently, an algorithm was published that allows for reconstructing the set point, even in an open-loop situation (20, 23, 24). The method is based on the minimal model of thyroid–pituitary interaction and on the observation that, in healthy volunteers, the set point is located in the region of the highest curvature of the pituitary response curve. It requires a minimum of two TSH–FT4 pairs, which were obtained with a latency of at least 4 weeks. Then, two parameters, *S* (multiplier) and φ (slope of exponential function), are determined, either algebraically or *via* regression, to fit the negative exponential function

$$\left[\text{TSH}\right]=Se^{-\varphi \left[\text{F}{\text{T}}_{4}\right]}$$

to the data. The next step is to find the root of the third derivative of the pituitary function, where the curvature

$$K=\frac{\varphi^{2}Se^{-\varphi \left[\text{F}{\text{T}}_{4}\right]}}{{\left(1+\varphi^{2}S^{2}e^{-2\varphi \left[\text{F}{\text{T}}_{4}\right]}\right)}^{{\;}^{3}/{\;}_{2}}}$$

is at its maximum. From this, the set point components for FT4 and TSH can be obtained with

$${\left[\text{F}{\text{T}}_{4}\right]}_{\text{SP}}=\frac{\ln\left(\varphi S\sqrt{2}\right)}{\varphi }$$

and

$${\left[\text{TSH}\right]}_{\text{SP}}=\frac{1}{\varphi \sqrt{2}}.$$

The algorithm has been validated in a small trial, which revealed in all examined cases a goodness-of-fit between 95 and 99% (20). It has still not been investigated, however, if a set point-based dose titration regime leads to a better QoL compared with the standard strategy on the premise of population-derived reference ranges.

## Closing Remarks and Future Perspectives

In this brief overview, we have described several calculated parameters derived from mathematical modeling that have emerged from recent clinical studies, as helpful tools in defining thyroid function. By extending the classical concept of separate measurements of thyroid hormone parameters these markers add new qualitative and quantitative dimensions to the evaluation of thyroid homeostasis.

Multivariate methods should improve diagnostic discrimination, as they account for interrelationships between thyroid parameters and permit determination of personal set points that are more narrowly defined than population-based reference ranges. Measuring conversion efficiency may particularly benefit the subgroup of patients with reduced QoL, despite normal TSH concentrations.

The use of structure parameters offers a more integrated and systemic view and has already delivered important insights into the physiology of pituitary–thyroid feedback control. Clinical applications are still experimental at present, and more trials are required to prove their utility for medical decision-making.

## Author Contributions

JD designed the validation study and developed Monte Carlo simulation software. JD, JM, and RH drafted the manuscript. GL-M, EW, HK, and AC recruited patients for formal evaluation of structure parameters. All authors read and approved the manuscript.

## Conflict of Interest Statement

JD received funding and personal fees from Sanofi-Henning, Hexal AG, and Pfizer and is the co-owner of the intellectual property rights for the patent “System and Method for Deriving Parameters for Homeostatic Feedback Control of an Individual” (Singapore Institute for Clinical Sciences, Biomedical Sciences Institutes, Application Number 201208940-5, WIPO number WO/2014/088516). All other authors declare that there is no conflict of interest that could be perceived as prejudicing the impartiality of the research reported.
