# Supplementary material for: Calculated Parameters of Thyroid Homeostasis: Emerging Tools for Differential Diagnosis and Clinical Research
Source: Front Endocrinol (Lausanne). 2016 Jun 9;7:57. doi: 10.3389/fendo.2016.00057 (PMC4899439; doi:10.3389/fendo.2016.00057)
Supplement: Supplementary file 1 [file datasheet_1.doc]

Supplementary Material

# Calculated parameters of thyroid homeostasis: Emerging tools for differential diagnosis and clinical research.

Johannes W. Dietrich*, Gabi Landgrafe-Mende, Evelin Wiora, Apostolos Chatzitomaris, Harald H. Klein, John E. M. Midgley, Rudolf Hoermann

*** Correspondence:** Corresponding Author: johannes.dietrich@ruhr-uni-bochum.de

# Supplementary Code

S Script simulating concentration-dependent coefficients of variations for hormone assays. This script links additive and multiplicative noise in order to simulate the combined effect of assay imprecision and biological variation. R 2.10 or newer required (R Project for Statistical Computing, RRID:SCR_001905).

# Monte Carlo simulation of concentration-dependent CVs of thyroid hormone assays #

# Version 2.1 (20160502)

# Total CVs according to published data:

# TSH (Clerico et al. 2015:

# 13.065% @ TSH = 0.5 mIU/l

# 11.653% @ TSH = 1.0 mIU/l

# 7.970% @ TSH = 10 mIU/l

# FT4 (Beckman Coulter data sheet B01902B):

# 9.2% @ FT4 = 4.6 ng/l (5.9 pmol/l)

# 4.95% @ FT4 = 7.6 ng/l (9.8 pmol/l)

# 4.32% @ FT4 = 20.4 ng/l (26.3 pmol/l)

# 5.05% @ FT4 = 42.7 ng/l (55.0 pmol/l)

# FT3 (Beckman Coulter data sheet A33734D):

# 10.4% @ FT3 = 1.4 pg/ml (2.2 pmol/l)

# 5.7% @ FT3 = 2,6 pg/ml (4.0 pmol/l)

# 5.3% @ FT3 = 9.6 pg/ml (14.8 pmol/l)

SAMPLES <- 50;

CV <- function(x, ...)

{

return(100 * sd(x, ...) / mean(x, ...));

}

TSH <- seq(from = 0, to = 10, by = 0.1);

FT3 <- seq(from = 2, to = 16, by = 0.14);

FT4 <- seq(from = 5, to = 60, by = 0.55);

CV.TSH <- seq(from = 0, to = 20, by = 0.2);

plot(TSH , CV.TSH, type="n", ylim = c(5, 30), main = "Compound noise explaining concentration-dependent CV", xlab = "Real TSH", ylab = "CV of measured TSH");

lines(TSH, 11.653 * TSH^-0.165, col = "red");

lines(TSH, 100*(0.07 + 0.061/TSH ), col = "blue");

legend("topright", legend = c("Clerico's equation", "Compound equation"), lwd = 1, col = c("red", "blue"));

measured.TSH.m <- matrix(nrow = SAMPLES, ncol = length(TSH));

measured.TSH.a <- matrix(nrow = SAMPLES, ncol = length(TSH));

measured.TSH.c <- matrix(nrow = SAMPLES, ncol = length(TSH));

measured.FT3.m <- matrix(nrow = SAMPLES, ncol = length(FT3));

measured.FT3.a <- matrix(nrow = SAMPLES, ncol = length(FT3));

measured.FT3.c <- matrix(nrow = SAMPLES, ncol = length(FT3));

measured.FT4.m <- matrix(nrow = SAMPLES, ncol = length(FT4));

measured.FT4.a <- matrix(nrow = SAMPLES, ncol = length(FT4));

measured.FT4.c <- matrix(nrow = SAMPLES, ncol = length(FT4));

for (i in 1:length(TSH))

{

multiplicative.noise <- rnorm(n = SAMPLES , mean = 1, sd = 0.07);

additive.noise <- rnorm(n = SAMPLES , mean = 0, sd = 0.061);

measured.TSH.m[,i] <- TSH[i] * multiplicative.noise;

measured.TSH.a[,i] <- TSH[i] + additive.noise;

measured.TSH.c[,i] <- TSH[i] * multiplicative.noise + additive.noise;

}

for (i in 1:length(FT3))

{

multiplicative.noise <- rnorm(n = SAMPLES , mean = 1, sd = 0.05);

additive.noise <- rnorm(n = SAMPLES , mean = 0, sd = 0.20);

measured.FT3.m[,i] <- FT3[i] * multiplicative.noise;

measured.FT3.a[,i] <- FT3[i] + additive.noise;

measured.FT3.c[,i] <- FT3[i] * multiplicative.noise + additive.noise;

}

for (i in 1:length(FT4))

{

multiplicative.noise <- rnorm(n = SAMPLES , mean = 1, sd = 0.05);

additive.noise <- rnorm(n = SAMPLES , mean = 0, sd = 0.45);

measured.FT4.m[,i] <- FT4[i] * multiplicative.noise;

measured.FT4.a[,i] <- FT4[i] + additive.noise;

measured.FT4.c[,i] <- FT4[i] * multiplicative.noise + additive.noise;

}

mat <- data.frame(id = 1:(SAMPLES * length(TSH)));

mat$TSH <- rep(TSH, each = SAMPLES);

mat$measured.TSH.m <- c(measured.TSH.m[,1:length(TSH)]);

mat$measured.TSH.a <- c(measured.TSH.a[,1:length(TSH)]);

mat$measured.TSH.c <- c(measured.TSH.c[,1:length(TSH)]);

mat$FT3 <- rep(FT3, each = SAMPLES);

mat$measured.FT3.m <- c(measured.FT3.m[,1:length(FT3)]);

mat$measured.FT3.a <- c(measured.FT3.a[,1:length(FT3)]);

mat$measured.FT3.c <- c(measured.FT3.c[,1:length(FT3)]);

mat$FT4 <- rep(FT4, each = SAMPLES);

mat$measured.FT4.m <- c(measured.FT4.m[,1:length(FT4)]);

mat$measured.FT4.a <- c(measured.FT4.a[,1:length(FT4)]);

mat$measured.FT4.c <- c(measured.FT4.c[,1:length(FT4)]);

mat$TSH.cat <- cut(mat$TSH, breaks = seq(from=0, to=11, by=0.1), include.lowest = TRUE, right = FALSE);

mat$FT3.cat <- cut(mat$FT3, breaks = seq(from=2, to=17, by=0.14), include.lowest = TRUE, right = FALSE);

mat$FT4.cat <- cut(mat$FT4, breaks = seq(from=5, to=61, by=0.55), include.lowest = TRUE, right = FALSE);

plot(mat$measured.TSH.m ~ mat$TSH, pch = 19, main = "Effect of multiplicative noise only", xlab = "Simulated real TSH", ylab = "Simulated measured TSH", col = "darkred");

plot(mat$measured.TSH.a ~ mat$TSH, pch = 19, main = "Effect of addititve noise only", xlab = "Simulated real TSH", ylab = "Simulated measured TSH", col = "darkred");

plot(mat$measured.TSH.c ~ mat$TSH, pch = 19, main = "Effect of combined additive and multiplicative noise", xlab = "Simulated real TSH", ylab = "Simulated measured TSH", col = "darkred");

plot(mat$measured.FT3.m ~ mat$FT3, pch = 19, main = "Effect of multiplicative noise only", xlab = "Simulated real FT3", ylab = "Simulated measured FT3", col = "darkgreen");

plot(mat$measured.FT3.a ~ mat$FT3, pch = 19, main = "Effect of addititve noise only", xlab = "Simulated real FT3", ylab = "Simulated measured FT3", col = "darkgreen");

plot(mat$measured.FT3.c ~ mat$FT3, pch = 19, main = "Effect of combined additive and multiplicative noise", xlab = "Simulated real FT3", ylab = "Simulated measured FT3", col = "darkgreen");

plot(mat$measured.FT4.m ~ mat$FT4, pch = 19, main = "Effect of multiplicative noise only", xlab = "Simulated real FT4", ylab = "Simulated measured FT4", col = "darkblue");

plot(mat$measured.FT4.a ~ mat$FT4, pch = 19, main = "Effect of addititve noise only", xlab = "Simulated real FT4", ylab = "Simulated measured FT4", col = "darkblue");

plot(mat$measured.FT4.c ~ mat$FT4, pch = 19, main = "Effect of combined additive and multiplicative noise", xlab = "Simulated real FT4", ylab = "Simulated measured FT4", col = "darkblue");

cat("CV in relation to TSH value categories:\n");

print(CV.cat <- tapply(mat$measured.TSH.c, mat$TSH.cat, CV));

plot(CV.cat, xaxt = "n", ylim = c(5, 30), pch = 19, main = "CV in relation to TSH value categories", xlab = "TSH class", ylab = "CV", col = "darkred");

axis(side = 1, at = 1:length(CV.cat), labels = names(CV.cat));

cat("\nCV in relation to FT3 value categories:\n");

print(CV.cat <- tapply(mat$measured.FT3.c, mat$FT3.cat, CV));

plot(CV.cat, xaxt = "n", ylim = c(5, 30), pch = 19, main = "CV in relation to FT3 value categories", xlab = "FT3 class", ylab = "CV", col = "darkgreen");

axis(side = 1, at = 1:length(CV.cat), labels = names(CV.cat));

cat("\nCV in relation to FT4 value categories:\n");

print(CV.cat <- tapply(mat$measured.FT4.c, mat$FT4.cat, CV));

plot(CV.cat, xaxt = "n", ylim = c(5, 30), pch = 19, main = "CV in relation to FT4 value categories", xlab = "FT4 class", ylab = "CV", col = "darkblue");

axis(side = 1, at = 1:length(CV.cat), labels = names(CV.cat));
