# Supplementary material for: Calculated Parameters of Thyroid Homeostasis: Emerging Tools for Differential Diagnosis and Clinical Research
Source: Front Endocrinol (Lausanne). 2016 Jun 9;7:57. doi: 10.3389/fendo.2016.00057 (PMC4899439; doi:10.3389/fendo.2016.00057)
Supplement: Supplementary file 2 [file table_1.doc]

Supplementary Material

# Calculated parameters of thyroid homeostasis: Emerging tools for differential diagnosis and clinical research.

Johannes W. Dietrich*, Gabi Landgrafe-Mende, Evelin Wiora, Apostolos Chatzitomaris, Harald H. Klein, John E. M. Midgley, Rudolf Hoermann

*** Correspondence:** Corresponding Author: johannes.dietrich@ruhr-uni-bochum.de

# Supplementary Table 1

De-identified data of patients in figure 1c. TI: Thyrotropic insufficiency; TT: Thyrotoxicosis; PH: Primary hypothyroidism; ST: Secondary hyperthyroidism. Parameters are reported with units of measurement and reference interval.

| No. | TSH (0.4–4.3 mIU/l) | FT4  (7.6–16.1 pmol/l) | FT3  (4.0–5.7 pmol/l) | SPINA-GT  (1.4–8.7 pmol/s) | SPINA-GD  (20–60 nmol/s) | Jostel's TSH index (1.3–4.1) | sTSHI {–2–+2) | TTSI  (<150) | Group |
| --- | --- | --- | --- | --- | --- | --- | --- | --- | --- |
| 1 | 0.87 | 7.7 | 4.4 | 2.44 | 52.69 | 0.90 | –2.66 | 26.87 | TI |
| 2 | 0.97 | 7.7 | 3.9 | 2.25 | 46.70 | 1.01 | –2.50 | 29.96 | TI |
| 3 | 1.09 | 7.7 | 4.2 | 2.07 | 50.29 | 1.12 | –2.33 | 33.67 | TI |
| 4 | 18.96 | 18.0 | 5.4 | 1.57 | 27.71 | 5.37 | 3.94 | 1366.49 | ST |
| 5 | 0.58 | 11.6 | 3.7 | 5.05 | 29.54 | 1.01 | –2.50 | 26.87 | TI |
| 6 | 1.00 | 6.4 | 4.2 | 1.83 | 60.35 | 0.87 | –2.71 | 25.74 | TI |
| 7 | 1.00 | 7.7 | 4.8 | 2.20 | 57.48 | 1.04 | –2.46 | 30.89 | TI |
| 8 | 0.13 | 3.9 | 3.8 | 6.49 | 91.00 | –1.52 | –6.24 | 2.01 | TI |
| 9 | 9.44 | 29.6 | 11.2 | 2.90 | 34.99 | 6.23 | 5.22 | 1117.73 | ST |
| 10 | 1.29 | 12.9 | 5.1 | 3.06 | 36.64 | 1.99 | –1.06 | 66.41 | normal |
| 11 | 1.77 | 10.3 | 5.2 | 2.00 | 46.70 | 1.96 | –1.10 | 72.90 | normal |
| 12 | 2.25 | 10.3 | 6 | 1.74 | 53.88 | 2.20 | –0.75 | 92.66 | normal |
| 13 | 1.19 | 10.3 | 5.2 | 2.59 | 46.70 | 1.56 | –1.69 | 49.01 | normal |

# Supplementary Table 1 (continued)

| No. | TSH (0.4–4.3 mIU/l) | FT4  (7.6–16.1 pmol/l) | FT3  (4.0–5.7 pmol/l) | SPINA-GT  (1.4–8.7 pmol/s) | SPINA-GD  (20–60 nmol/s) | Jostel's TSH index (1.3–4.1) | sTSHI {–2–+2) | TTSI  (<150) | Group |
| --- | --- | --- | --- | --- | --- | --- | --- | --- | --- |
| 14 | 1.98 | 7.7 | 4.6 | 1.40 | 55.08 | 1.72 | –1.45 | 61.16 | normal |
| 15 | 0.67 | 11.6 | 5.4 | 4.49 | 43.11 | 1.16 | –2.28 | 31.04 | normal |
| 16 | 0.46 | 12.9 | 4.8 | 6.82 | 34.49 | 0.95 | –2.58 | 23.68 | normal |
| 17 | 0.25 | 18.0 | 3.2 | 16.41 | 16.42 | 1.04 | –2.46 | 18.02 | TT |
| 18 | 0.01 | 33.5 | NA | 701.08 | NA | –0.10 | –4.15 | 1.34 | TT |
| 19 | 90.66 | 3.9 | 3.2 | 0.30 | 76.63 | 5.03 | 3.44 | 1400.15 | PH |
| 20 | 0.09 | 63.1 | 12.8 | 151.06 | 18.77 | 6.07 | 4.99 | 22.70 | TT |
| 21 | 63.73 | 6.4 | 4.1 | 0.51 | 58.91 | 5.02 | 3.43 | 1640.41 | PH |
| 22 | 0.01 | 61.8 | 32.8 | 1294.30 | 49.10 | 3.70 | 1.48 | 2.47 | TT |
| 23 | 0.03 | 51.5 | 17.1 | 362.13 | 30.72 | 3.42 | 1.06 | 6.18 | TT |
| 24 | 0.04 | 52.8 | 28.1 | 279.39 | 49.24 | 3.88 | 1.74 | 8.44 | TT |
| 25 | 0.01 | 41.2 | 15.9 | 862.86 | 35.70 | 0.93 | –2.61 | 1.65 | TT |
| 26 | 0.03 | 45.0 | 27.2 | 316.87 | 55.84 | 2.55 | –0.22 | 5.41 | TT |
| 27 | 22.98 | 6.4 | 3.7 | 0.55 | 53.16 | 4.00 | 1.92 | 591.51 | PH |
| 28 | 76.32 | 5.1 | 4.2 | 0.40 | 75.44 | 5.03 | 3.44 | 1571.58 | PH |
| 29 | 0.02 | 25.7 | 6.5 | 270.62 | 23.35 | –0.45 | –4.66 | 2.06 | TT |
| 30 | 86.8 | 3.9 | 2.1 | 0.30 | 50.29 | 4.98 | 3.38 | 1340.54 | PH |
| 31 | 0.04 | 23.2 | 5.8 | 122.66 | 23.15 | –0.10 | –4.15 | 3.71 | TT |
| 32 | 22.26 | 7.7 | 3.9 | 0.66 | 46.70 | 4.14 | 2.13 | 687.57 | PH |
| 33 | 0.02 | 51.5 | 19.9 | 541.24 | 35.75 | 3.01 | 0.46 | 4.12 | TT |
| 34 | 0.02 | 32.2 | 10.5 | 338.28 | 30.18 | 0.42 | –3.38 | 2.57 | TT |
| 35 | 0.09 | 43.8 | 18.3 | 104.82 | 38.67 | 3.48 | 1.15 | 15.75 | TT |
| 36 | 0.02 | 55.3 | 10.8 | 581.84 | 18.05 | 3.53 | 1.23 | 4.43 | TT |
| 37 | 0.01 | 20.6 | 8.4 | 431.43 | 37.72 | –1.84 | –6.71 | 0.82 | TT |
| 38 | 0.02 | 36.0 | 9.2 | 378.87 | 23.61 | 0.93 | –2.61 | 2.88 | TT |
| 39 | 0.03 | 32.2 | 6.6 | 226.33 | 18.97 | 0.82 | –2.78 | 3.86 | TT |
| 40 | 0.01 | 21.9 | 6.6 | 458.40 | 27.89 | –1.66 | –6.45 | 0.88 | TT |
| 41 | 0.01 | 20.6 | 8.5 | 431.43 | 38.17 | –1.84 | –6.71 | 0.82 | TT |

# Supplementary Table 1 (continued)

| No. | TSH (0.4–4.3 mIU/l) | FT4  (7.6–16.1 pmol/l) | FT3  (4.0–5.7 pmol/l) | SPINA-GT  (1.4–8.7 pmol/s) | SPINA-GD  (20–60 nmol/s) | Jostel's TSH index (1.3–4.1) | sTSHI {–2–+2) | TTSI  (<150) | Group |
| --- | --- | --- | --- | --- | --- | --- | --- | --- | --- |
| 42 | 0.54 | 10.3 | 5 | 4.76 | 44.90 | 0.77 | –2.86 | 22.24 | TI |
| 43 | 1.26 | 6.4 | 4.6 | 1.55 | 66.10 | 1.10 | –2.37 | 32.43 | TI |
| 44 | 0.34 | 10.3 | 5.2 | 7.10 | 46.70 | 0.31 | –3.54 | 14.00 | TI |
| 45 | 1.37 | 6.4 | 4.2 | 1.47 | 60.35 | 1.18 | –2.25 | 35.26 | TI |
| 46 | 0.82 | 6.4 | 3.6 | 2.13 | 51.73 | 0.67 | –3.01 | 21.11 | TI |
| 47 | 0.17 | 6.4 | 4 | 8.39 | 57.47 | –0.91 | –5.33 | 4.38 | TI |
